# Failed back surgery syndrome after non-instrumented lumbar surgery: Clinical report of a Tertiary care neurosurgical center

**DOI:** 10.12669/pjms.42.5.14717

**Published:** 2026-05

**Authors:** Zahid Khan, Seema Sharafat, Muhammad Sohaib Khan, Syed Jawad Ahmad

**Affiliations:** 1 Zahid Khan, FCPS, Department of Neurosurgery, MTI Lady reading Hospital, Peshawar, Pakistan; 2 Seema Sharafat, FCPS, Department of Neurosurgery, MTI Lady reading Hospital, Peshawar, Pakistan; 3 Muhammad Sohaib Khan, MBBS, Department of Neurosurgery, MTI Lady reading Hospital, Peshawar, Pakistan; 4 Syed Jawad Ahmad, MBBS, Department of Neurosurgery, MTI Lady reading Hospital, Peshawar, Pakistan

**Keywords:** Failed back surgery syndrome, Laminectomy, Low back pain, Lumbar decompression, Recurrent disc herniation

## Abstract

**Objective:**

This study aimed to determine the frequency, etiological spectrum, postoperative pain and functional outcomes of Failed Back Surgery Syndrome (FBSS) among patients undergoing non-instrumented lumbar decompression at a tertiary care neurosurgical center.

**Methodology:**

A retrospective cohort study was conducted at the Department of Neurosurgery, Lady Reading Hospital, Peshawar, from July 2021 to June 2025. Patients aged ≥ 15 years who had lumbar decompression without instrumentation because of degenerative spinal disease were enrolled, and those with congenital malformation, tumor, fusion, or premature loss to follow-up were excluded. A 12-month postoperative evaluation was then used to diagnose FBSS. Independent t-tests and chi-square tests were used to compare FBSS and non-FBSS groups and predictors of FBSS were determined using multivariable logistic regression. The Visual Analog Scale (VAS) and Oswestry Disability Index (ODI) were used to measure pain and functional disability.

**Results:**

Of 1,233 patients analyzed, 172 (13.9%) developed FBSS. The mean age was 48.51 ± 12.05 years, and 56.9% were male. No significant association was observed with age, BMI, gender, smoking, or depression (p > 0.05). The most frequent etiologies were recurrent disc herniation (36.0%), inadequate decompression (23.8%), and postoperative discitis (22.7%). FBSS patients had significantly higher pain and disability scores compared to non-FBSS patients (VAS 6.07 ± 1.79 vs. 2.09 ± 1.24; ODI 59.07 ± 10.40 vs. 22.15 ± 6.89; both p < 0.001).

**Conclusion:**

FBSS was found in 14% patients after non-instrumented lumbar decompression. Recurrent herniation, incomplete decompression, and postoperative infection were the leading causes. Patients with FBSS demonstrated significantly higher pain intensity and functional disability compared with those without FBSS.

## INTRODUCTION

Low back pain (LBP) is one of the leading health burdens in the world, and the only most common cause of disability worldwide. In 2021, the prevalence of LBP was estimated at 628.8 million (95% uncertainty interval [UI] 551.8-700.8 million).^1^ Other recent investigations have identified 452.8 million incidences of LBP in working-age adults, and an increase of 52.7 percent since 1990 with significant regional and sex disparities.^2^ In Pakistan, studies consistently report prevalence rates between 52% and 74% across various professional groups.^3^,^4^ It is well established that, although most of the LBP cases can be managed with conservative treatment, approximately 10 percent or more of the patients develop symptoms that persist after three months, thereby becoming chronic LBP.

Among LBP patients undergoing lumbar spine surgery, the condition known as Failed Back Surgery Syndrome (FBSS) also termed postsurgical spine syndrome or persistent spinal pain syndrome Type-II (PSPS-II) has emerged as a substantial challenge. FBSS is generally defined as the continuing or recurring back and/or leg pain subsequent to spinal surgery, or the inability of the procedure to provide satisfaction to the surgeon and relief to the patient.^5^,^6^ A recent systematic review calculated a pooled prevalence of about 14.97% (5-27.6) in post-spine surgery patients.^7^ It has been indicated by other sources that 20 percent of lumbar surgery patients are at risk of developing FBSS.^8^

The growing burden of FBSS is driven by multiple factors, increased numbers of spinal surgeries in ageing populations, expanding surgical indications, and the complex interplay of biological, surgical, and psychosocial risk determinants. Because it is refractory, the FBSS treatment may often require multimodal measures such as pharmacologic (NSAIDs, opioids, anticonvulsants), formal physical rehabilitation, psychosocial, and innovative neuromodulation (spinal cord stimulation) approaches. In fact, recent meta-analyses indicate better results in cases of neuromodulation in relation to conventional medical management in a selected cohort.^9^,^10^ In light of the heterogeneous etiologies and high rates of functional impairment associated with FBSS, better local data are urgently needed to inform targeted prevention and treatment strategies.

Although there is an emerging interest in FBSS as a significant complication following lumbar spine surgery, the majority of available evidence on the prevalence, the etiological range, and the clinical consequences are based on research in high-income countries. There is still limited data from the developing regions like Pakistan. Therefore, locally collected data play a crucial role in the understanding of the trends and clinical implications of FBSS in local healthcare institutions. Thus, the present study was conducted to determine the frequency, etiological spectrum, and postoperative pain and functional outcomes associated with FBSS among patients undergoing non-instrumented lumbar decompression at a tertiary care neurosurgical center in Pakistan.

## METHODOLOGY

This retrospective observational cohort study was conducted at the Department of Neurosurgery, Medical and Teaching Institute (MTI), Lady Reading Hospital, Peshawar, over a four-year period from July 2021 to June 2025.

### Ethical approval:

It was obtained from the Institutional Review Board of MTI, Lady Reading Hospital, Peshawar (Ref No. 462/LRH/MTI; Dated August 26, 2025). Written informed consent was obtained from all participants prior to inclusion. Patient confidentiality was maintained throughout the study in accordance with the Declaration of Helsinki (2013 revision). Data were de-identified, coded, and stored securely with access restricted to the principal investigators.

Consecutive sampling was used to reduce selection bias and ensure that all eligible patients were included in the analysis. No formal sample size calculation was carried out. Instead, all consecutive eligible patients during the defined time frame were included to maximize statistical power and enhance the external validity of the findings.

### Inclusion criteria:

All consecutive patients aged above 15 years, of either gender, who underwent lumbar spine surgery without instrumentation for degenerative spinal disease during the study period were eligible for inclusion.

### Exclusion criteria:

Patients were excluded in case they had surgery on either, congenital spinal malformations, spinal malignancies or redo (revision) surgery, or they had spinal fusion because of instability. Patients who did not have adequate clinical records or incomplete follow-up data were also excluded from the analysis.

The measured variables were demographic data (age and gender), clinical diagnosis, type and degree of degenerative lumbar pathology, surgical specifics (type and length of operation), and post-operative outcomes. The primary outcome measure was the occurrence of FBSS, which can be described as the occurrence of persistent or recurrent back and/or leg pain with functional limitation ≥12 months after surgery, consistent with contemporary definitions of Persistent Spinal Pain Syndrome Type-II (PSPS-II). The secondary outcomes were postoperative pain, functional (status) and the return-to-work status. Patients with congenital or malignant spinal conditions were excluded in order to ensure homogeneity and minimize confounding.

Retrospective data collection was undertaken based on hospital medical records, operative notes, and follow up clinic documentation. Appropriate preoperative, intraoperative and postoperative data were collected using a structured data collection proforma.

The diagnosis of Failed Back Surgery Syndrome (FBSS) was based solely on the final outcome assessment conducted at 12 months postoperatively, in accordance with accepted definitions requiring persistent or recurrent symptoms.

FBSS and the etiologies associated with it were diagnosed through a combination of clinical assessment, radiological assessment, and laboratory testing. The postoperative imaging was plain radiography (X-ray) and magnetic resonance imaging (MRI) to assess recurrent disc herniation and the adequacy of decompression. In the suspected postoperative discitis patients, clinical, MRI, and inflammatory laboratory markers, especially high erythrocyte sedimentation rate (ESR) were used to make the diagnosis.

The severity of the pain was measured by means of Visual Analog Scale (VAS),^11^ and functional status by the Oswestry Disability Index (ODI).^12^ Both VAS and ODI are validated and extensively utilized instruments in the measurement of postoperative recovery and quality of life after spinal surgery.

### Statistical analysis:

All data were analyzed using IBM SPSS Statistics version 23.0. Demographic, clinical and operative characteristics were summarized using descriptive statistics. To facilitate the analytical approach, patients were divided into two categories according to the postoperative outcome, including those who experienced Failed Back Surgery Syndrome (FBSS) and those who did not (non-FBSS). These two groups were then compared to determine demographic factors, clinical variables, and postoperative outcomes. Independent sample t-tests were employed to compare continuous variables whereas chi-square tests were used to test categorical variables. The independent variables with a p-value of less than 0.10 in the univariate analysis were incorporated into a multivariate logistic regression model to determine the independent predictors of FBSS. Each risk factor was calculated to have adjusted odds ratios (ORs) with 95 percent confidence intervals (CIs). The p-value of less than 0.05 was considered statistically significant. The severity of pain and functional outcomes assessed using VAS and ODI scores respectively were compared in FBSS and non-FBSS groups. Postoperative functional recovery was also compared by using chi-square analysis to obtain the return-to-work rates.

## RESULTS

As shown in Table-I, out of 1,233 patients included in the study, 172 (13.9%) developed FBSS. The mean age was 48.55 ± 12.14 years in the non FBSS group and 48.28 ± 11.58 years in the FBSS group (p = 0.782) and BM (28.38 ± 3.88 vs. 28.42 ± 4.09 kg/m²; p = 0.897)were similar between FBSS and non-FBSS groups. Gender, smoking status, depression, and surgical procedure type (laminectomy vs. microdiscectomy) were not significantly associated with FBSS (all p > 0.05). However, return-to-work status showed a strong relationship, as only 27.9% of FBSS patients resumed work compared with 89.5% of non-FBSS patients (p < 0.001).

**Table-I:** Association Between Categorical Variables and Failed Back Surgery Syndrome (FBSS) (N = 1233).

Variables		FBSS		Total (n=1233)	p-value
		No (n=1061)	Yes (n=172)		
Age (years)		48.55 ± 12.14	48.28 ± 11.58	48.51±12.05	0.782
BMI (kg/m²)		28.42 ± 4.09	28.38 ± 3.88	28.41±4.05	0.897
Gender	Female	466 (43.9%)	66 (38.4%)	532 (43.1%)	0.173
	Male	595 (56.1%)	106 (61.6%)	701 (56.9%)	
Smoker	No	820 (77.3%)	138 (80.2%)	958 (77.7%)	0.389
	Yes	241 (22.7%)	34 (19.8%)	275 (22.3%)	
Depression	No	978 (92.2%)	159 (92.4%)	1137 (92.2%)	0.904
	Yes	83 (7.8%)	13 (7.6%)	96 (7.8%)	
Procedure	Laminectomy	304 (28.7%)	49 (28.5%)	353 (28.6%)	0.965
	Microdiscectomy	757 (71.3%)	123 (71.5%)	880 (71.4%)	
Returned to Work	No	111 (10.5%)	124 (72.1%)	235 (19.1%)	< 0.001*
	Yes	950 (89.5%)	48 (27.9%)	998 (80.9%)	

Pearson’s χ² or t-test as appropriate;

* p < 0.05 significant.

**Fig.1:**
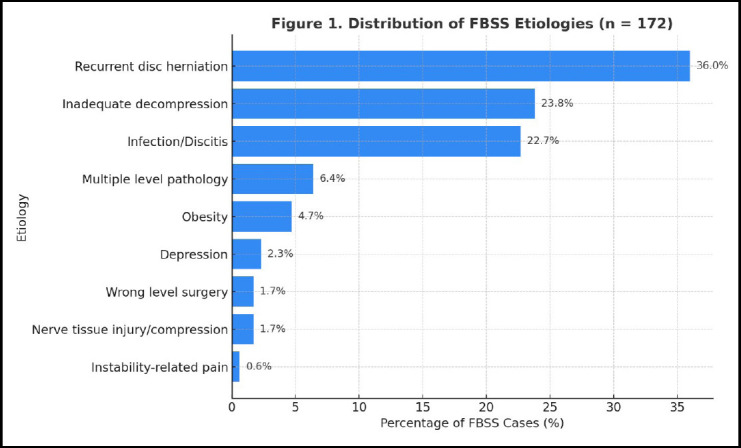
Distribution of FBSS etiologies.

The etiological distribution presented in Fig.1 indicates that recurrent disc herniation (36.0%), inadequate decompression (23.8%), and postoperative infection or discitis (22.7%) were the most frequent causes of FBSS. Less common factors included multiple-level pathology, obesity, depression, and wrong-level surgery. Upon stratification by surgical procedure, both laminectomy and microdiscectomy showed similar patterns of FBSS etiology. Recurrent disc herniation remained the most common cause in both groups, followed by inadequate decompression and postoperative infection. There was no statistically significant difference between the etiological distribution and surgical procedures (p = 0.458).

Pain and disability scores, summarized in Table-II, were significantly higher in FBSS patients. The mean VAS score was 6.07 ± 1.79 in the FBSS group versus 2.09 ± 1.24 in non-FBSS patients (p < 0.001), while the mean ODI score was 59.07 ± 10.40 versus 22.15 ± 6.89, respectively (p < 0.001), reflecting greater pain intensity and functional limitation in FBSS cases.

**Table-II:** Comparison of Pain and Functional Disability Scores Between FBSS and Non-FBSS Groups (N = 1233).

Variable	FBSS Group	Mean ± SD	Median (IQR)	Mean Rank	Z	p-value
VAS Score	No	2.09 ± 1.24	2.10 (1.40 - 3.00)	537.50	−19.48	< 0.001*
	Yes	6.07 ± 1.79	6.20 (4.90 - 7.50)	1107.42		
ODI Score	No	22.15 ± 6.89	22.10 (17.20 - 26.60)	531.17	−21.02	< 0.001*
	Yes	59.07 ± 10.40	58.30 (51.10 - 63.70)	1146.47		

Mann-Whitney U test;

* p < 0.05 significant.

Multivariate logistic regression results in Table-III revealed that demographic and perioperative factors such as age, BMI, gender, smoking, depression, and procedure type were not independent predictors of FBSS.

**Table-III:** Binary Logistic Regression Predicting Failed Back Surgery Syndrome (FBSS).

Predictors	Model 1	p-value
	OR (95 % CI)	
Age (years)	0.998 (0.985–1.012)	0.780
BMI (kg/m²)	1.254 (0.901–1.745)	0.180
Male (vs Female)	0.997 (0.958–1.038)	0.897
Smoker (Yes vs No)	0.841 (0.562–1.259)	0.401
Depression (Yes vs No)	0.955 (0.519–1.757)	0.883
Microdiscectomy (vs Laminectomy)	0.982 (0.686–1.405)	0.919

Binary logistic regression (enter method); OR = Odds Ratio; CI = Confidence Interval; Model fit: χ²(6) = 2.705, p = 0.845; Nagelkerke R² = 0.004.

## DISCUSSION

In the present study, the incidence of FBSS was 13.9% (172 out of 1,233 patients), which is consistent with contemporary global estimates. Recent reviews place the prevalence of FBSS or its updated term *Persistent Spinal Pain Syndrome Type-2 (PSPS-T2)* between 10% and 46%, with a pooled mean of approximately 14.97%.^7^,^13^ In 2024, a multicenter study also found similar results, stating that 15-20% of patients who have undergone lumbar surgery have persistent or recurrent pain.^14^

The multifactorial etiology of FBSS has been reaffirmed by recent literature, which has been classified in to preoperative, intraoperative, or postoperative.^5^,^15^ Modifiable patient variables that contribute to preoperative factors are obesity, smoking, and untreated psychological illnesses. Technical errors like wrong-level exposure or incomplete decompression are the intraoperative etiologies, and the etiologies of the postoperative are recurrent disc herniation, infection, epidural fibrosis, or adjacent-segment degeneration. Psychosocial factors especially depression and anxiety are also factors that significantly contribute to the perceived pain and postoperative satisfaction.^16^,^17^ In the present study, the most common cause of FBSS was recurrent disc herniation, which was found in 62 patients (36.0%), followed by inadequate decompression in 41 patients (23.8%), and postoperative discitis in 39 patients (22.7%). A large surgical case series reported that inadequate decompression accounted for approximately 20-30% of FBSS cases.^18^ Likewise Dhatt et al., emphasizes inadequate decompression, misdiagnosis, iatrogenic injury, and psychosocial contributors across 95 surgeries.^19^ Furthermore Xu et al., identified hypertension, intermittent claudication, high-intensity zones, and Modic changes as significant predictor of FBSS in 333 patients.^20^

The difference in postoperative pain and disability between FBSS and non-FBSS patients was another substantial finding of this study. Individuals who had FBSS had a much higher VAS pain score (6.07 ± 1.79 vs. 2.09 ± 1.24) and ODI disability score (59.07 ± 10.40 vs. 22.15 ± 6.89) than those who did not develop FBSS. Elkholy et al., explicitly states FBSS is characterized by the presence of persistent or recurring low back pain that has a significant impact on patients’ quality of life.^21^ Yeo et al., similarly describes FBSS as resulting in persistent or recurrent pain and “reduced quality of life.^22^ A retrospective analysis of 44 patients Cipollina et al., documented significant disability using the ODI (49.0 baseline), with pain measured on VAS (8 baseline).^23^

Recent reviews emphasize that the incidence of FBSS is rising worldwide in parallel with the growing volume of spinal operations. A 2023 risk-factor modeling study found that posterior open lumbar surgery significantly increases FBSS risk, especially in patients with preoperative comorbidities or suboptimal intraoperative visualization.^14^ However, age, BMI, smoking status, and depression were not found to be significant predictors of FBSS in the present cohort, indicating that postoperative and intraoperative factors might have had a more significant role in the development of FBSS in our patient group.

Special attention should be paid to psychological health. Though the prevalence of depression was only reported at 7.6% in FBSS cases in this study, its actual prevalence is probably much greater since most psychological symptoms are not well diagnosed. In line with the previous evidence, untreated depression plays a major role in worsening surgical outcomes and satisfaction.^24^ Psychosocial distress preoperative screening and addressing patient expectations thus becomes very important factors of surgical planning and postoperative management.

Even though management techniques were not directly assessed in the given study, the available literature indicates in favor of a multimodal approach to FBSS management and the use of pharmacological therapy, rehabilitation, and interventional pain procedures. The conservative management usually entails analgesics, neuropathic pain drugs and designed physiotherapy programs. Neuromodulation therapy (Spinal cord stimulation-SCS) has provided promising results in patients with refractory symptoms, with multiple systematic reviews reporting greater long-term pain management and functional activity than conventional medical management.^25^ Latest technological advances (high-frequency and multicolumn spinal cord stimulation systems) have further enhanced treatment outcomes in patients with persistent postoperative spinal pain.

### Strengths of the study:

The present study has several strengths. The comparatively large sample was suitable for a systematic evaluation of the demographic variables, surgical variables, and postoperative functional outcomes such as pain severity and disability. Additionally, the research offers valuable regional information, where little has been published in the evidence on FBSS. Nevertheless, further research is needed to better understand long-term outcomes and optimal management strategies for FBSS. To enhance prevention strategies and postoperative care, multicenter designs, increased follow-up periods, and standardized psychological testing, and careful analysis of treatment methods should be introduced into future studies.

### Limitations:

The major limitation of the study was the limited duration of postoperative follow-ups, which probably lacked the ability to detect delayed etiologies like fibrosis or adjacent-segment disease. Secondly, instrumented and oncologic cases were excluded, thus interfering with generalization of the results to more complex spinal groups. Another limitation might be the under-identification of psychosocial factors. Though the status of depression was measured based on clinical history, no standardized psychological assessment tools (such as Patient Health Questionnaire-9 -PHQ-9 or Beck Depression Inventory-BDI) were utilized in the collection of data. Thus, there is a possibility that the prevalence of depression in this cohort is underestimated.

## CONCLUSION

Failed Back Surgery Syndrome (FBSS) remains a relatively frequent complication following lumbar decompression surgery. FBSS was found in 172 of 1233 patients (14.0%). The most common etiologies were recurrent disc herniation (62/172; 36.0%), inadequate decompression (41/172; 23.8%), and postoperative discitis (39/172; 22.7%). Patients that developed FBSS had a significantly higher postoperative pain and functional disability than those who did not. The results of the study further indicate that FBSS has significant clinical effects and that postoperative assessment is essential in patients undergoing lumbar surgery.

### Author’s Contribution:

**ZK, SJA:** Conceived, designed and did statistical analysis & editing of manuscript, is responsible for integrity of research.

**MSK:** Did data collection and manuscript writing.

**SS:** Did review and final approval of manuscript.
